# Supplementary material for: Generation of realistic synthetic data using Multimodal Neural Ordinary Differential Equations
Source: NPJ Digit Med. 2022 Aug 20;5:122. doi: 10.1038/s41746-022-00666-x (PMC9391444; doi:10.1038/s41746-022-00666-x)
Supplement: Supplementary file 1 — Supplementary Material [file 41746_2022_666_MOESM1_ESM.pdf]

Supplementary Material: Generation of realistic synthetic data using multimodal neural ordinary differential equations

Philipp Wendland‡[1,2], Colin Birkenbihl‡[1,3], Marc Gomez-Freixa[3], Meemansa Sood[1,3], Maik Kschischo[2], Holger Fröhlich*[1,3]

‡ Authors contributed equally to this work.

1. Department of Bioinformatics, Fraunhofer Institute for Algorithms and Scientific Computing (SCAI), Sankt Augustin 53754, Germany
2. Department of Mathematics and Technology, University of Applied Sciences Koblenz, Remagen 53424, Germany
3. Bonn-Aachen International Center for IT, Rheinische Friedrich-Wilhelms-Universität Bonn, Bonn 53115, Germany

***Corresponding Author:**

Holger Fröhlich

holger.froehlich@scai.fraunhofer.de

Fraunhofer-Institute for Algorithms and Scientific Computing (SCAI)

Schloss Birlinghoven

D-53757 Sankt Augustin

Supplementary Results

Supplementary Table 1: Summary statistic of marginal distributions shown in Figure 2 in the main text.

	Mean (SD)	25 %	Median	75%	JS-Div.
**SCOPA month 12**					
Real	0.28 (1.11)	-0.52	0.12	0.92	-
Posterior	0.27 (0.94)	-0.42	0.18	0.76	0.01
VAMBN	0.31 (1.08)	-0.42	0.31	0.99	0.02
Prior	0.41 (0.8)	-0.06	0.46	0.89	0.03
**UPDRS2 month 24**					
Real	0.73 (1.34)	-0.15	0.59	1.33	-
Posterior	0.66 (1.23)	-0.17	0.41	1.37	0.01
VAMBN	0.7 (1.17)	-0.13	0.66	1.52	0.02
Prior	0.87 (1.17)	0.06	0.88	1.64	0.03
**A-beta 42 static**					
Real	0.0 (1.0)	-0.65	-0.05	0.55	-

Posterior	0.01 (1.2)	-0.71	-0.11	0.54	0.01
VAMBN	0.02 (0.93)	-0.65	0.03	0.6	0.01
Prior	-069 (3.72)	-1.65	-0.52	0.77	0.01

Additional figures for PPMI experiments

To provide an overview about more variables, we present additional plots equivalent to those in the main text below. Plots for all variables, including relevant summary statistics like those presented in Supplementary Table 1, can be found under: https://github.com/philippwendland/MultiNODEs

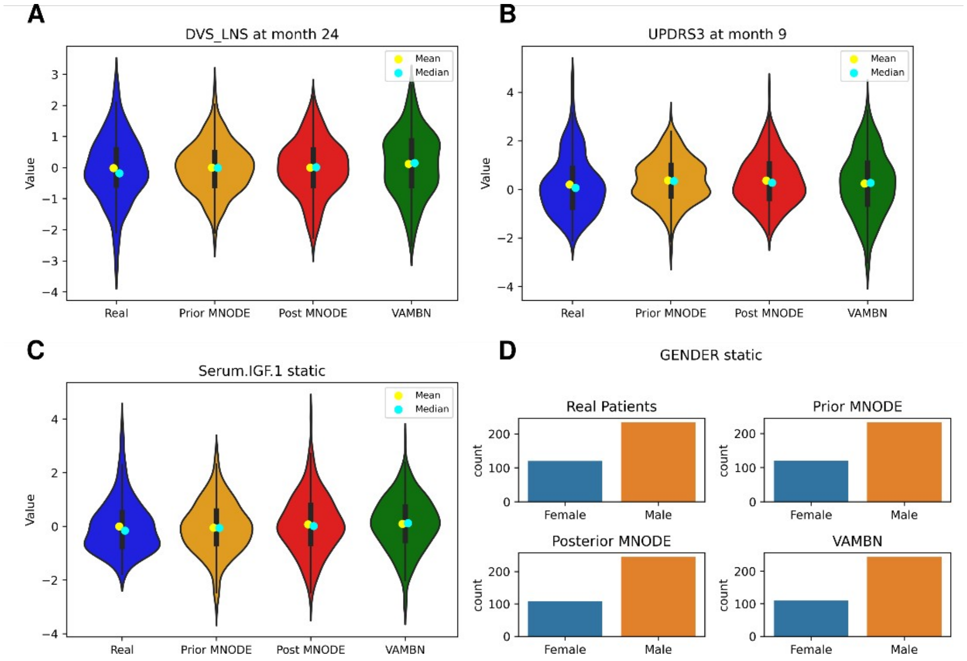

Supplementary Figure 1: Marginal distributions encountered in the generated synthetic data and real data of PPMI. Comprises additional examples corresponding to Figure 2 in the main text. Similar figures for all variables can be found at: https://github.com/philippwendland/MultiNODEs/tree/main/Plots/PPMI/Synthetic_Data_Generation/Static_violin

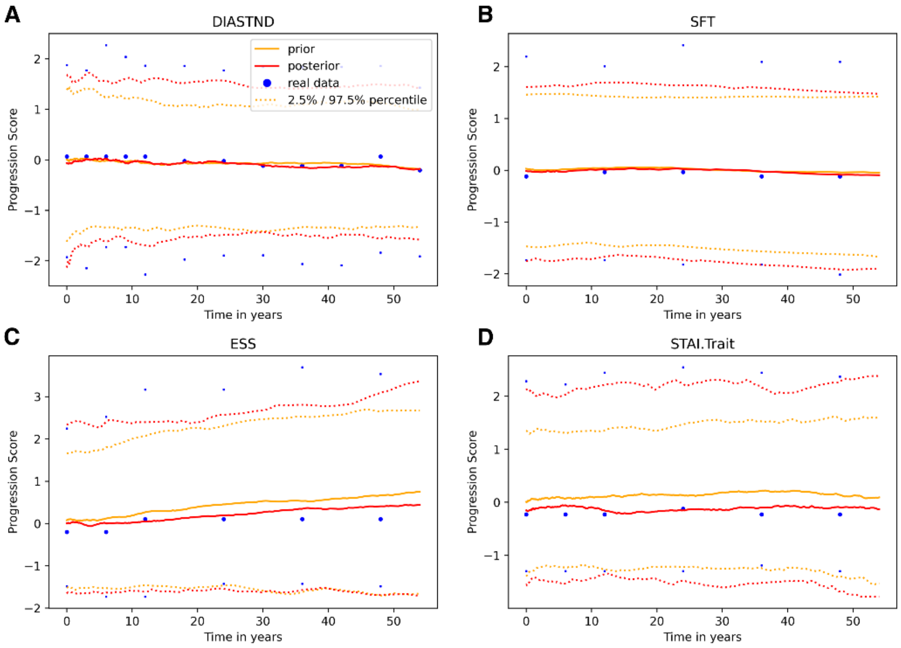

Supplementary Figure 2: Comparison of median trajectories including 2.5% / 97.5% quantiles of longitudinal variables from synthetic and real PPMI data. Comprises additional examples corresponding to Figure 3 in the main text. Similar figures for all variables can be found under: https://github.com/philippwendland/MultiNODEs/tree/main/Plots/PPMI/Synthetic_Data_Generation/Generated_Median.

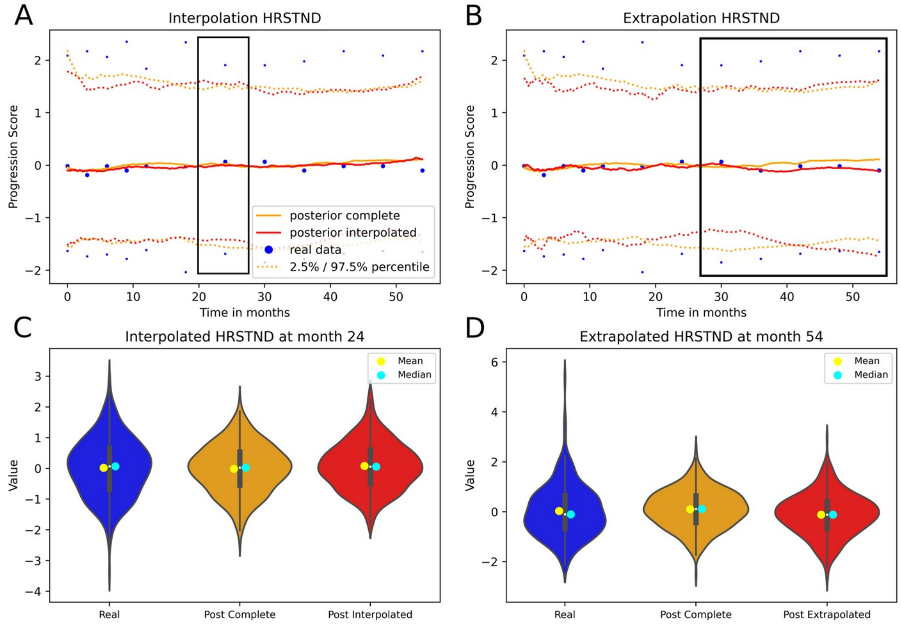

Supplementary Figure 3: Time-continuous interpolation and extrapolation of exemplary PPMI variables. The black box indicates the interpolated and extrapolated sections. Comprises additional examples corresponding to Figure 3 in the main text. Similar figures for all variables can be found under:

https://github.com/philippwendland/MultiNODEs/tree/main/Plots/PPMI. **A,** interpolation of the HRSTND variable at month 24. **B,** extrapolation of the last two assessments of the variable. **C,** marginal distribution of the interpolated values at visit 24. **D,** marginal distribution of the extrapolated values at month 48.

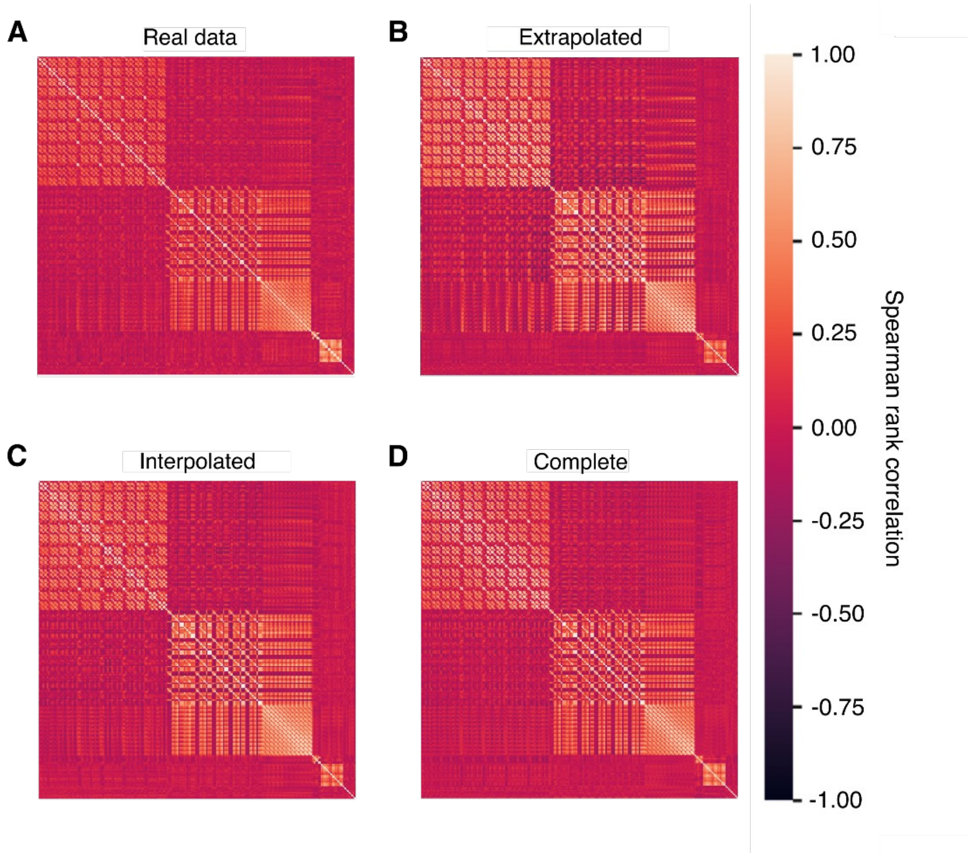

Supplementary Figure 4: Correlation structure of the interpolated and extrapolated PPMI data (i.e., generated using only parts of the real data) in comparison to the correlation structure of the real data and that of synthetic data generated based on the complete real data.

NACC results

Below, we present the results based on the NACC dataset. All displayed results originate from experiments that were equivalent to those presented in the main text for the PPMI data. Figures for all experiments and all variables can be found under https://github.com/philippwendland/MultiNODEs/tree/main/Plots/NACC.

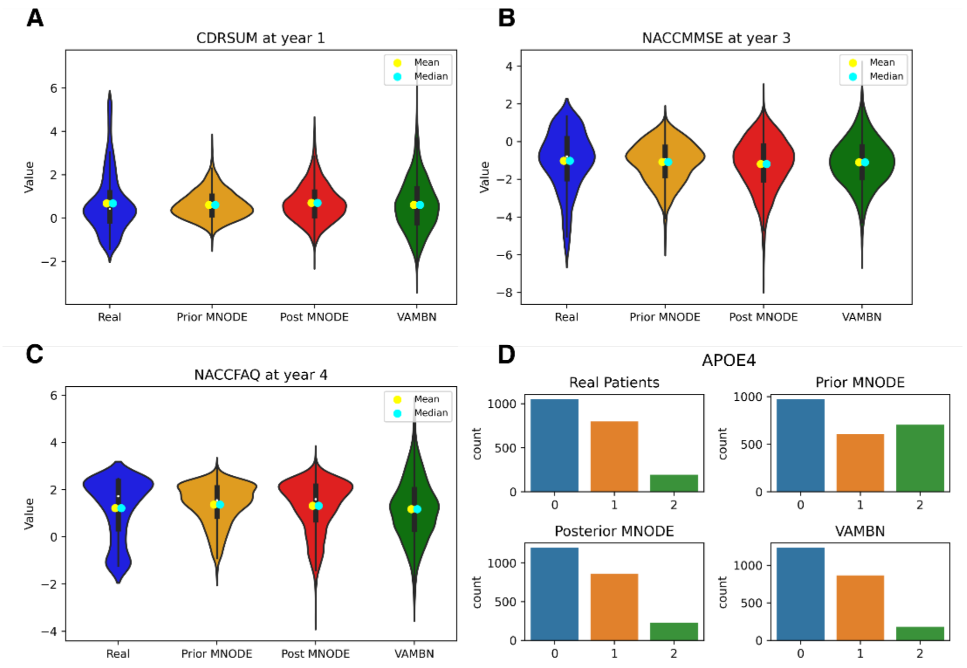

Supplementary Figure 5: Marginal distributions of real and synthesized data for multiple variables of the NACC data. Mean, standard deviation and KL-Divergence for the displayed variables can be found and similar figures for all NACC variables can be found under: https://github.com/philippwendland/Multimodal_Neural_ODEs/tree/main/Plots/NACC.

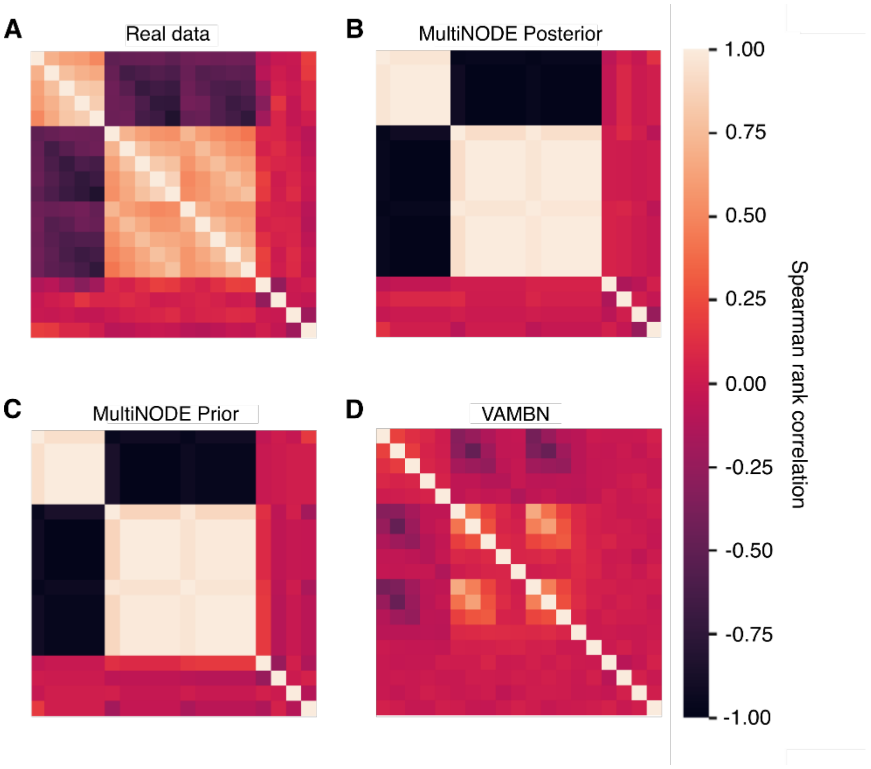

Supplementary Figure 6: Correlation structure of real and synthetic NACC data expressed as spearman rank correlation coefficients. **A,** real data. **B,** posterior sampling from MultiNODEs. **C,** prior sampling from MultiNODEs. **D,** VAMBN generated data.

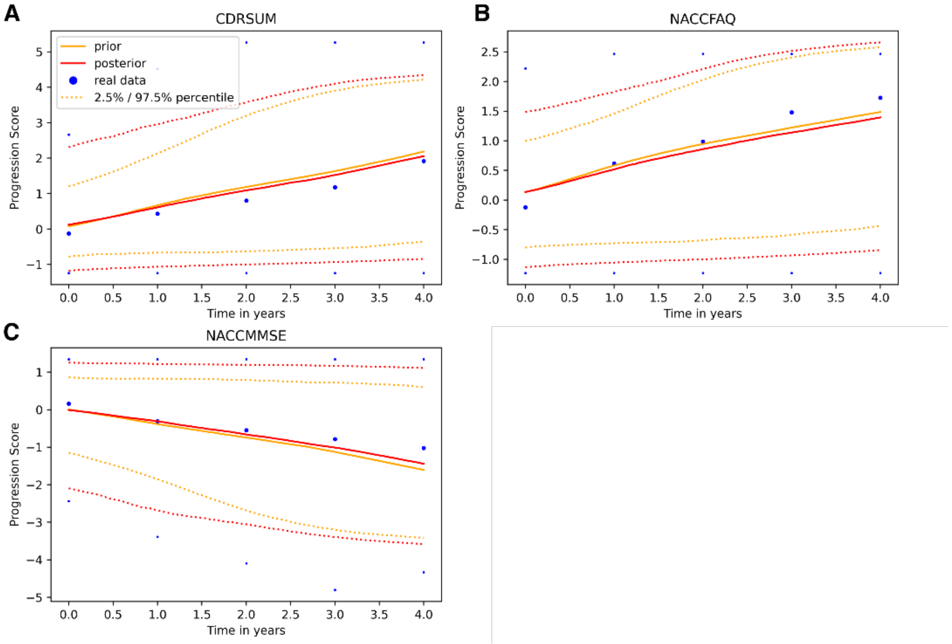

Supplementary Figure 7: Comparison of median trajectories including the 2.5% / 97.5% quantiles of longitudinal variables from synthetic and real NACC data. **A, B, C,** depict different longitudinal variables from the NACC dataset.

Interpolation of the NACC data was performed for year 2. For extrapolation, MultiNODEs were trained on data up to year 2 and the values for years 3 and 4 were extrapolated. Exemplary results are shown in **Supplementary Figure 8**. We observed that the mean JS-divergence calculated across all variables between the interpolated data and the real data was slightly higher (0.064 ± 0.054) than that of the real data and the synthetic data generated after training MultiNODEs on the complete trajectory (0.049 ± 0.026).

As in the interpolation setting, we again compared the average JS-divergence between the extrapolated data and the real data with that between the real data and synthetic data that were generated after training MultiNODEs on the complete trajectory. As

expected, we could see a larger difference between the JS-divergences compared to the interpolation setting with 0.065 ± 0.024 for the extrapolated data and 0.022 ± 0.009 for the synthetic data based on the complete trajectory.

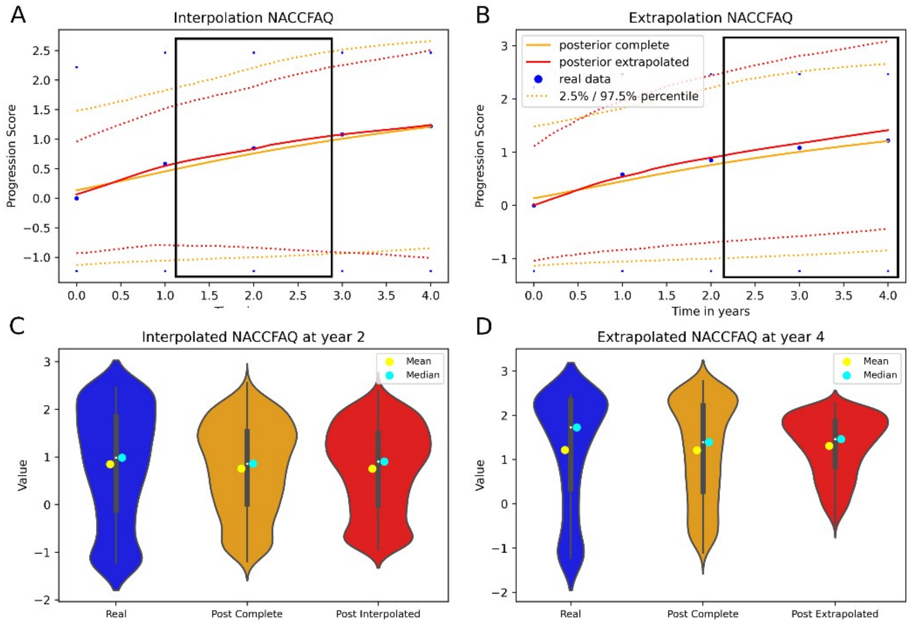

Supplementary Figure 8: Time-continuous interpolation and extrapolation of NACC variables. The black box indicates the interpolated and extrapolated sections. Similar figures for all variables can be found under: https://github.com/philippwendland/MultiNODEs/tree/main/Plots/NACC. **A,** interpolation of the FAQ variable at year 2. **B,** extrapolation of the last two assessments of the FAQ variable. **C,** distribution of the interpolated values for FAQ at year 2. **D,** distribution of the extrapolated values for FAQ at year 4.

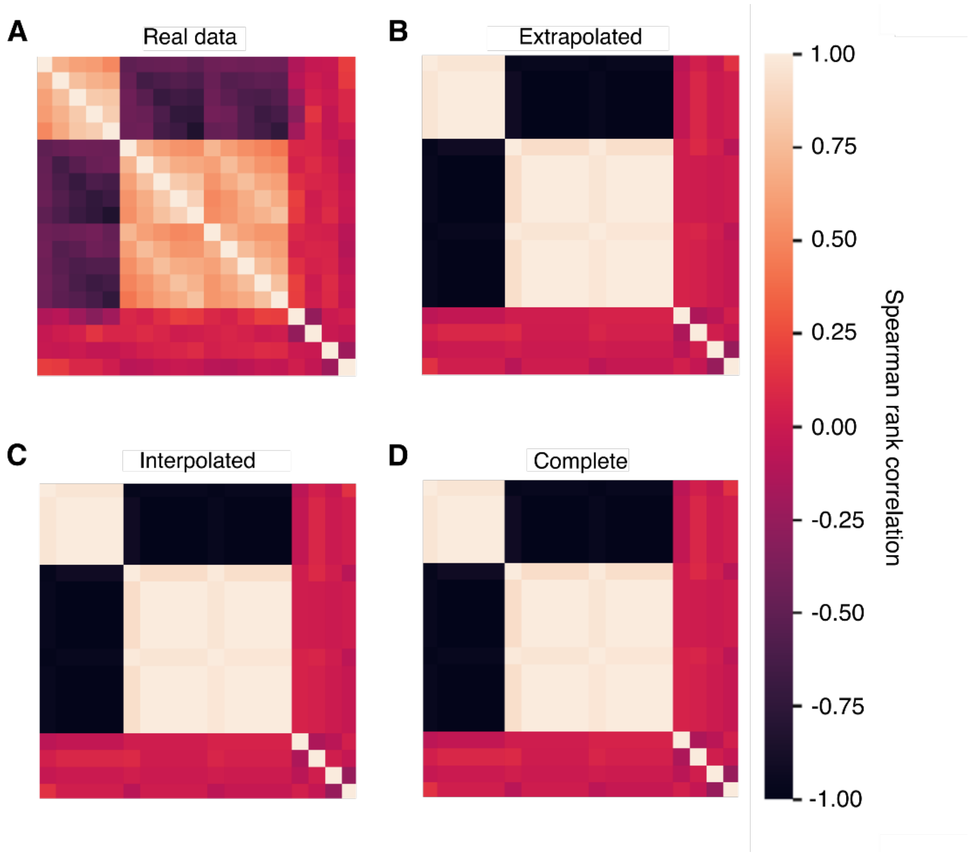

Supplementary Figure 9: Correlation structure of the interpolated and extrapolated NACC data (i.e., generated using only parts of the real data) in comparison to the correlation structure of the real data and that of synthetic data generated based on the complete real data.

Supplementary Methods

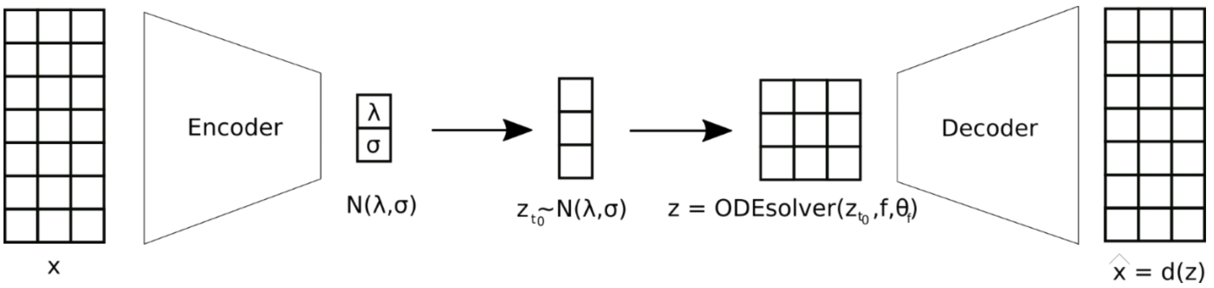

Supplementary Figure 10: Schema of a Neural ODE trained as a generative latent time series model

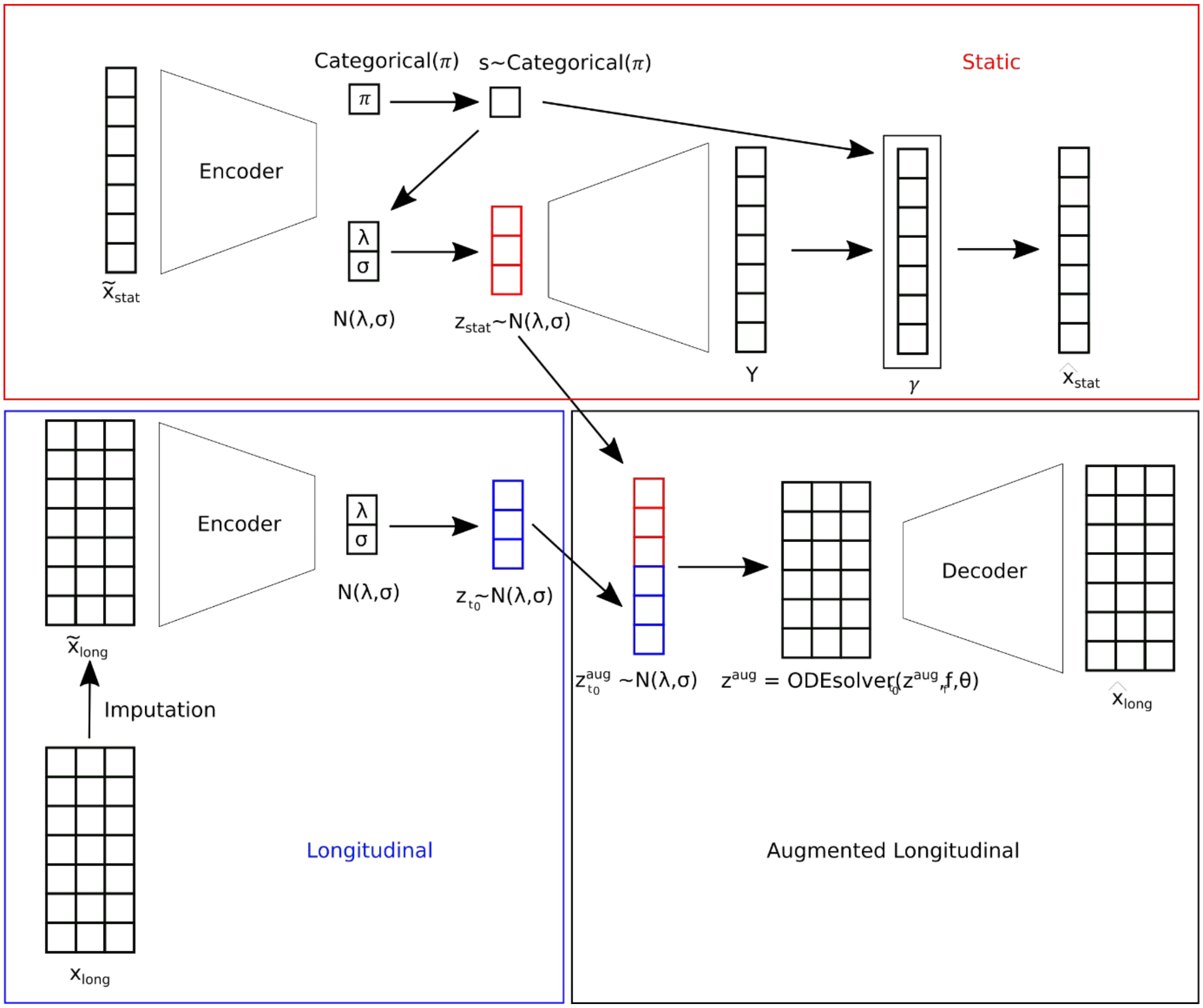

Supplementary Figure 11: Architecture of the MultiNODEs.

Hyperparameter optimization

To apply MultiNODEs (and the VAMBN) to data, it is necessary to perform hyperparameter optimization. In this work, we used a Bayesian hyperparameter optimization [1] as implemented in the Optuna package [2]. We used the default settings employing a Tree of Parzen Estimator.

The target function to minimize was the weighted Mean Squared Error of the longitudinal data as reconstruction loss validated by a 5-fold-cross-validation. We used early stopping in every fold with a delta of 0 and a patience of 50 epochs. To determine the optimal number of epochs for the final model, we computed the average number of epochs across the folds. For the SIR model experiments, a simple train-test split was performed instead of a cross-validation due to the large sample sizes of the simulated data. The following hyperparameters were considered (the final hyperparameter configurations per model are presented at the end of this document):

Learning rate	$[10^{-4}, 10^{-2}]$
Number of epochs	{100, 200, …, 4000}
Batch size as fraction of the number of patients	[0, 1]
Encoder for longitudinal variables	Elman-network or LSTM
Number of hidden units of the encoder in percentage of the product of number of time points and number of longitudinal variables	[0, 1]
In the case of an Elman-network as an encoder: Activation function of the hidden layers	Tanh, ReLU or identity function
Dimension of initial condition (ignoring static features) of the latent ODE in percent of the number of longitudinal variables	[0, 4]
Number of hidden units of the feed-forward network representing the right-hand side of the latent ODE in percentage of the dimension of the initial conditions (ignoring static features) of the Neural latent ODE	[0, 10]
Steps to solve in the latent ODE	{1, 2, …, 8}
Activation function of the feed-forward network representing the right-hand side of the latent ODE	Tanh, ReLU, identity function
Number of hidden units in the decoder network, expressed as percentage of the product of number of timepoints and the number of longitudinal variables	[0, 1]
Activation function of the decoder	Tanh, ReLU, identity function
Fraction of input drop-out units in the decoder	[0, 1]
Number of mixture components in the Gaussian Mixture Model	{1, 2, …, 10}
Dimension of the latent representation of the static variables	{1,2, …,10}
Weighting parameter in MultiNODE ELBO	[0, 2]

Supplementary Figure 12 shows a hyperparameter optimization run on the simulated SIR data.

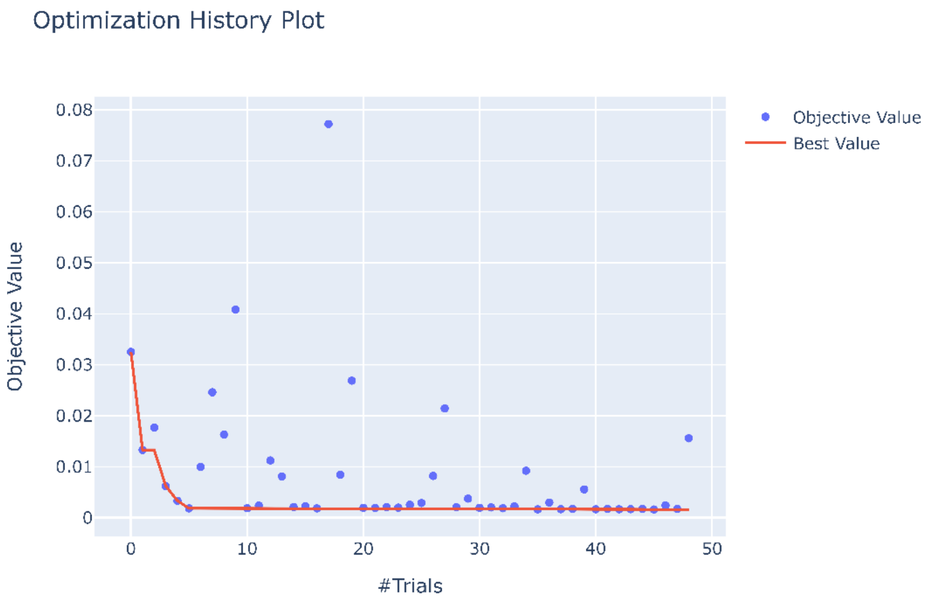

Supplementary Figure 12: Trials of a hyperparameter optimization run performed on the SIR data.

To train the VAMBN approach, it is again necessary to conduct a hyperparameter optimization. For every module of VAMBN, we performed a Bayesian hyperparameter optimization. We evaluated every configuration of hyperparameters with a 5-fold-cross-validation using the reconstruction loss (cross entropy) as a target function. The hyperparameters were the following:

Learning rate	$[10^{-4}, 10^{-2}]$
Mini-batch size	{16, 32}
Number of epochs	{200, …, 7000}

SIR Model

As parameters for the SIR model, we set T equal to 40, beta equal to 2, and gamma equal to 1. The population size was set to 1000.

Classifier training

We used the random forest implementation of sklearn with their default hyperparameters. To impute missing values in the real data, we used the missForest iterative imputer of sklearn for a maximum of 100 iterations with 10 estimators.

Supplementary Figure 13 shows the relative feature importance for the respective classifiers. Feature importance was calculated as decrease in classifier performance when the respective feature was excluded. For PPMI, the feature importance was accurately reflected in all synthetic classifiers. For NACC, we mainly saw lower importance of biological sex across all synthetic dataset-trained classifiers and an increase in importance for FAQ, a functional assessment of patients. Conclusively, the feature importance was quite stable between synthetic and real data-trained classifiers for both datasets.

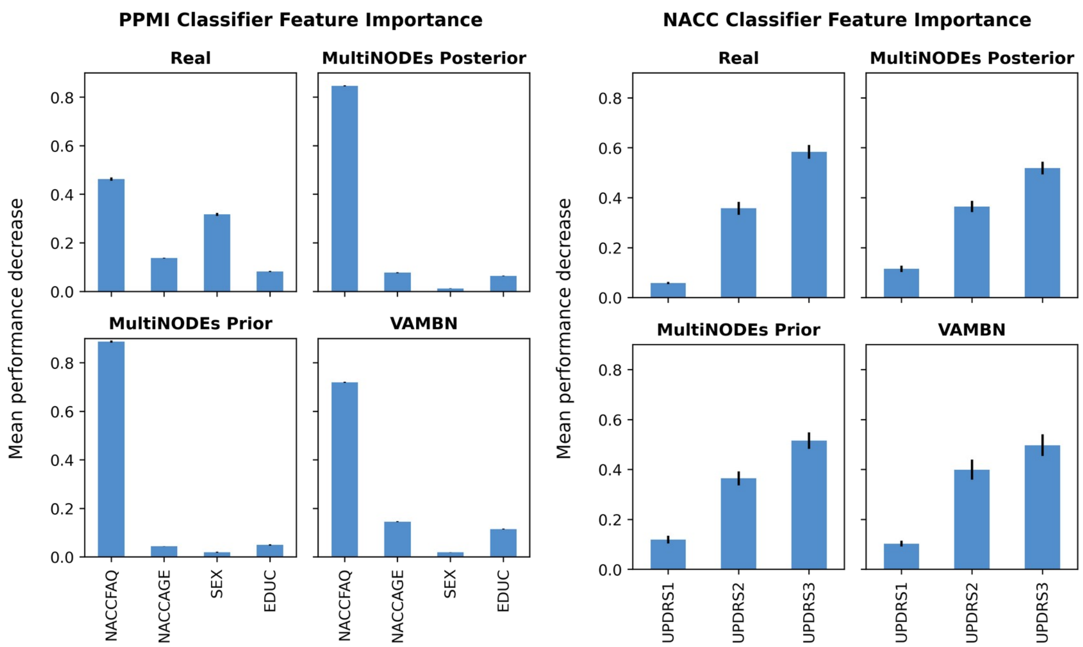

Supplementary Figure 13: Feature importance for predictors used to discriminate between real healthy control subjects and real and synthetic patients, respectively. Error bars depict the standard deviation calculated over 10 repeated runs of each classifier.

Preprocessing of the PPMI data

To express the disease progression, we used z-scores normalized to a patient's baseline values. Let $x^n_{t_i, j_{long}}$ be the value of a longitudinal variable j_{long} at a time point t_i of patient n, $\mu_{t_{bl}, j_{long}}$ be the mean of a longitudinal variable at baseline and $\sigma_{t_{bl}, j_{long}}$ be the standard deviation of that variable at baseline. Then a z-score with respect to baseline can be defined as follows

$$\frac{x^n_{t_i, j_{long}} - \mu_{t_{bl}, j_{long}}}{\sigma_{t_{bl}, j_{long}}}$$

The static real valued variables were standardized, and the static categorical variables one-hot encoded.

To handle SNPs, we use CADD-filtered Impact Scores and Polygenetic Risk Scores. We receive odds ratios for the risk SNPs from the Phenome Wide Association Studies (PheWAS) catalog of genome-wide association studies (GWAS) [3]. To compute a polygenetic risk score for every patient, we sum up the odds ratio of the occurring risk SNPs per patient. The so-called Combined Annotation Dependent Depletion (CADD) values rate the maleficence of SNPs. To get CADD-filtered impact scores, we sum up the number of occurring risk SNPs over the recommended threshold of 15 per patient.

List of Variables in PPMI

All longitudinal variables of the modules Medical History and UPDRS are measured at month 0, 3, 6, 9, 12, 18, 24, 30, 36, 42, 48, and 54. All longitudinal variables of the Non-Motor Module are measured at month 0, 12, 24, 36, and 48 (the following variables are measured at month 6th too: ESS, GDS, Quip, RBD, SCOPA, STAI.State, STAI.Trait, and STA). CSF-Alpha-Synuclein is measured at month 0, 6, 12, 24, 36.

**Variable**	**Module**	**Name / Explanation**
WGTKG	Medical History	Weight in kilograms
HTCM	Medical History	Height in centimeters
TEMPC	Medical History	Temperature in Celsius
SYSSUP	Medical History	Systolic blood pressure (supine position)
DYSSUP	Medical History	Diastolic blood pressure (supine position)
HRSUP	Medical History	Heart frequency (supine position)
SYSTNP	Medical History	Systolic blood pressure (standing position)
DYSSTNP	Medical History	Diastolic blood pressure (standing position)
HRSTNP	Medical History	Heart frequency (standing position)
DVT-Total recall	Non-Motor	Subscore of Hopkins Verbal Learning Test
DVS-LNS	Non-Motor	Letter number sequencing
ESS	Non-Motor	Epworth‘s Sleepiness Scale
SCOPA	Non-Motor	Scales for outcomes in PD-autonomic
SFT	Non-Motor	Semantic fluency
STA	Non-Motor	State trait anxiety total score
STAI.State	Non-Motor	STAI – State subscore

STAI.Trait	Non-Motor	STAI – Trait subscore
MDS-UPDRS 1	UPDRS	Non-Motor values of daily living
MDS-UPDRS 2	UPDRS	Motor values of daily living
MDS-UPDRS 3	UPDRS	Motor examinations
CSF-Alpha-Synuclein		Cerebrospinal fluid alpha-synuclein

Supplementary Table 2: Longitudinal variables of the de-novo-patients (with modules of VAMBN)

Variable	Module	Datatype	Name / explanation
A-beta42	Biological	Numerical	Beta-amyloid 42 [39]
pTau	Biological	Numerical	Phospho-tau
Tau	Biological	Numerical	Total-tau
Tau/A-beta	Biological	Numerical	Total-tau/A42
PTau/A-beta	Biological	Numerical	Phospho-tau/A-beta42
pTau.Tau	Biological	Numerical	Phospho-tau/Total-tau
ALDH1A1..rep.1	Biological	Numerical	Aldehyde dehydrogenase 1-family
ALDH1A1..rep.2	Biological	Numerical	Aldehyde dehydrogenase 1-family
GAPDH..rep.1	Biological	Numerical	Glyceraldehyde 3-phosphate dehydrogenase
GAPDH..rep.2	Biological	Numerical	Glyceraldehyde 3-phosphate dehydrogenase
HSPA8..rep.1	Biological	Numerical	Heat shock 70 kDa protein 8
HSPA8..rep.2	Biological	Numerical	Heat shock 70 kDa protein 8
LAMB2..rep.1	Biological	Numerical	Laminin subunit beta-2
LAMB2..rep.2	Biological	Numerical	Laminin subunit beta-2
PGK1..rep.1	Biological	Numerical	Phosphoglycerate kinase 1
PGK1..rep.2	Biological	Numerical	Phosphoglycerate kinase 1
PSMC4..rep.1	Biological	Numerical	26S protease regulatory subunit 6B
PSMC4..rep.2	Biological	Numerical	26S protease regulatory subunit 6B
SKP1..rep.1	Biological	Numerical	Guanine nucleotide exchange factor SPIKE 1
SKP1..rep.2	Biological	Numerical	Guanine nucleotide exchange factor SPIKE 1
UBE2K..rep.1	Biological	Numerical	Ubiquitin-conjugating enzyme E2 K
UBE2K..rep.2	Biological	Numerical	Ubiquitin-conjugating enzyme E2 K
Serum.IGF.1	Biological	Numerical	Insulin-like growth factor 1
RAINDALS	Demographic	Categorical	Indians and indigenious americans
RAASIAN	Demographic	Categorical	Asians
RABLACK	Demographic	Categorical	Afroamericans
RAWHITE	Demographic	Categorical	White
RANOS	Demographic	Categorical	Not-specified ethnicity
EDUCYRS	Demographic	Numerical	Years of school education
HANDED	Demographic	Categorical	Handedness
Gender	Demographic	Categorical	Gender
BIOMOMPD	Family illness	Categorical	Biological mother has PD
BIODADPD	Family illness	Categorical	Biological father has PD
FULSIBPD	Family illness	Categorical	Biological siblings have PD

MAGPARPD	Family illness	Categorical	Maternal grandparents have PD
PAGPARPD	Family illness	Categorical	Paternal grandparents have PD
MATAUPD	Family illness	Categorical	Maternal aunts and uncles have PD
PATAUPD	Family illness	Categorical	Paternal aunts and uncles have PD
Imaging		Categorical	DaTSCAN-scintigraphy
ENROLL AGE		Numerical	Age at baseline
CADD filtered impact scores		Numerical	Compare section…
Polygenetic risk scores		Numerical	Compare section…

Supplementary Table 3: Static variables of the PPMI de-novo PD patients.

NACC Variables

NACC's variables longitudinal variables were assessed annually over a period of up to 4 years.

NACCMMSE	Longitudinal	Numerical	Mini-Mental State Examination
CDRSUM	Longitudinal	Numerical	Clinical Dementia Rating scale Sum of Boxes
NACCFAQ	Longitudinal	Numerical	Functional Activities Questionnaire
NACCNE4S	Static	Categorical	APOE E4 status
NACCAGE	Static	Numerical	Patient age at study baseline
SEX	Static	Categorical	Biological sex
EDUC	Static	Categorical	Years of education

Supplementary Table 4: Variable description of the NACC study

Final model specifications

MultiNODEs implement several approaches to counteract overfitting to the training data. We implemented drop-out layers in the encoder, used cross-validation to tune hyperparameters, and the ELBO of the variational autoencoder framework represents another form of model regularization.

The models were optimized using the default implementation of pyTorch's Adam optimizer.

Below you find the chosen hyperparameters for all MultiNODEs used in the manuscript.

PPMI Model Hyperparameters

Learning rate	0.0015680290621642827
Number of epochs	1900
Batch size as fraction of the number of patients	0.6400910825235765
Encoder for longitudinal variables	Elman-network
Number of hidden units of the encoder in percentage of the product of number of time points and number of longitudinal variables	0.49866725741363976
In the case of an Elman-network as an encoder: Activation function of the hidden layers	Identity
Dimension of initial condition (ignoring static features) of the latent ODE in percent of the number of longitudinal variables	1.8837462054054002
Number of hidden units of the feed-forward network representing the right-hand side of the latent ODE in percent of the dimension of the initial conditions (ignoring static features) of the Neural latent ODE	4.584466407303982
Steps to solve in the latent ODE	1
Activation function of the feed-forward network representing the right-hand side of the latent ODE	Identity
Number of hidden units in the decoder network, expressed as percent of the product of number of timepoints and the number of longitudinal variables	0.6974659448314735
Activation function of the decoder	Identity
Fraction of input drop-out units in the decoder	0.4951111384939868
Number of mixture components in the Gaussian Mixture Model	6
Dimension of the latent representation of the static variables	3
Weighting parameter in MultiNODE ELBO	0.9675595125571386
Number of trainable parameters	129373

NACC Model Hyperparameters

Learning rate	0.0233168
Number of epochs	141
Batch size as fraction of the number of patients	0.656582
Encoder for longitudinal variables	LSTM
Number of hidden units of the encoder in percentage of the product of number of time	3.03908

points and number of longitudinal variables	
In the case of an Elman-network as an encoder: Activation function of the hidden layers	
Dimension of initial condition (ignoring static features) of the latent ODE in percent of the number of longitudinal variables	1.43289
Number of hidden units of the feed-forward network representing the right-hand side of the latent ODE in percent of the dimension of the initial conditions (ignoring static features) of the Neural latent ODE	4.00991
Steps to solve in the latent ODE	3
Activation function of the feed-forward network representing the right-hand side of the latent ODE	Identity
Number of hidden units in the decoder network, expressed as percent of the product of number of timepoints and the number of longitudinal variables	4.09659
Activation function of the decoder	ReLU
Fraction of input drop-out units in the decoder	0.0378523
Number of mixture components in the Gaussian Mixture Model	14
Dimension of the latent representation of the static variables	8
Weighting parameter in MultiNODE ELBO	1.60768
Number of trainable parameters	22241

SIR standard

Learning rate	0.00678686
Number of epochs	676
Batch size as fraction of the number of patients	0.367031
Encoder for longitudinal variables	Elman-network
Number of hidden units of the encoder in percentage of the product of number of time points and number of longitudinal variables	0.436512
In the case of an Elman-network as an encoder: Activation function of the hidden layers	TanH
Dimension of initial condition (ignoring static features) of the latent ODE in percent of the number of longitudinal variables	1.04597
Number of hidden units of the feed-forward network representing the right-hand side of the latent ODE in percent of the dimension of the initial conditions (ignoring static features) of the Neural latent ODE	3.79528
Steps to solve in the latent ODE	5
Activation function of the feed-forward network representing the right-hand side of the latent ODE	Identity
Number of hidden units in the decoder network, expressed as percent of the product of number of	0.910399

timepoints and the number of longitudinal variables	
Activation function of the decoder	TanH
Fraction of input drop-out units in the decoder	0.00180114
Number of mixture components in the Gaussian Mixture Model	2
Dimension of the latent representation of the static variables	6
Weighting parameter in MultiNODE ELBO	1.3888
Number of trainable parameters	2035

SIR n=100

Learning rate	0.00601775920584171
Number of epochs	579
Batch size as fraction of the number of patients	0.3567612219833565
Encoder for longitudinal variables	Elman-network
Number of hidden units of the encoder in percentage of the product of number of time points and number of longitudinal variables	0.7504019762037379
In the case of an Elman-network as an encoder: Activation function of the hidden layers	ReLU
Dimension of initial condition (ignoring static features) of the latent ODE in percent of the number of longitudinal variables	1.010561217834188
Number of hidden units of the feed-forward network representing the right-hand side of the latent ODE in percent of the dimension of the initial conditions (ignoring static features) of the Neural latent ODE	3.6777847577609126
Steps to solve in the latent ODE	4
Activation function of the feed-forward network representing the right-hand side of the latent ODE	Identity
Number of hidden units in the decoder network, expressed as percent of the product of number of timepoints and the number of longitudinal variables	0.9328043779498616
Activation function of the decoder	ReLU
Fraction of input drop-out units in the decoder	0.048294395239418614
Number of mixture components in the Gaussian Mixture Model	1
Dimension of the latent representation of the static variables	6
Weighting parameter in MultiNODE ELBO	0.6323358754903038
Number of trainable parameters	2670

SIR N=5000

Learning rate	0.00998745
Number of epochs	1513
Batch size as fraction of the number of patients	0.17319
Encoder for longitudinal variables	Elman-network
Number of hidden units of the encoder in percentage of the product of number of time points and number of longitudinal variables	0.226659
In the case of an Elman-network as an encoder: Activation function of the hidden layers	Identity
Dimension of initial condition (ignoring static features) of the latent ODE in percent of the number of longitudinal variables	0.196854
Number of hidden units of the feed-forward network representing the right-hand side of the latent ODE in percent of the dimension of the initial conditions (ignoring static features) of the Neural latent ODE	1.59344
Steps to solve in the latent ODE	1
Activation function of the feed-forward network representing the right-hand side of the latent ODE	TanH
Number of hidden units in the decoder network, expressed as percent of the product of number of timepoints and the number of longitudinal variables	0.904631
Activation function of the decoder	Identity
Fraction of input drop-out units in the decoder	0.0515771
Number of mixture components in the Gaussian Mixture Model	6
Dimension of the latent representation of the static variables	2
Weighting parameter in MultiNODE ELBO	1.35137
Number of trainable parameters	424

SIR t=5

Learning rate	0.00828768
Number of epochs	546
Batch size as fraction of the number of patients	0.683492
Encoder for longitudinal variables	Elman-network
Number of hidden units of the encoder in percentage of the product of number of time points and number of longitudinal variables	0.649597
In the case of an Elman-network as an encoder: Activation function of the hidden layers	TanH
Dimension of initial condition (ignoring static features) of the latent ODE in percent of the number of longitudinal variables	0.479475
Number of hidden units of the feed-forward network representing the right-hand side of the latent ODE in percent of the dimension of the initial conditions (ignoring static features) of the	0.812782

Neural latent ODE	
Steps to solve in the latent ODE	5
Activation function of the feed-forward network representing the right-hand side of the latent ODE	Identity
Number of hidden units in the decoder network, expressed as percent of the product of number of timepoints and the number of longitudinal variables	0.857999
Activation function of the decoder	TanH
Fraction of input drop-out units in the decoder	0.00545472
Number of mixture components in the Gaussian Mixture Model	2
Dimension of the latent representation of the static variables	4
Weighting parameter in MultiNODE ELBO	0.633321
Number of trainable parameters	507

SIR t=100

Learning rate	0.00715729
Number of epochs	1480
Batch size as fraction of the number of patients	0.276469
Encoder for longitudinal variables	Elman-network
Number of hidden units of the encoder in percentage of the product of number of time points and number of longitudinal variables	0.00971514
In the case of an Elman-network as an encoder: Activation function of the hidden layers	Identity
Dimension of initial condition (ignoring static features) of the latent ODE in percent of the number of longitudinal variables	2.69398
Number of hidden units of the feed-forward network representing the right-hand side of the latent ODE in percent of the dimension of the initial conditions (ignoring static features) of the Neural latent ODE	0.515954
Steps to solve in the latent ODE	3
Activation function of the feed-forward network representing the right-hand side of the latent ODE	TanH
Number of hidden units in the decoder network, expressed as percent of the product of number of timepoints and the number of longitudinal variables	0.400573
Activation function of the decoder	ReLU
Fraction of input drop-out units in the decoder	0.173605
Number of mixture components in the Gaussian Mixture Model	6
Dimension of the latent representation of the static variables	2
Weighting parameter in MultiNODE ELBO	1.19349

Number of trainable parameters	2520

SIR noise 50%

Learning rate	0.00523903
Number of epochs	220
Batch size as fraction of the number of patients	0.831626
Encoder for longitudinal variables	LSTM
Number of hidden units of the encoder in percentage of the product of number of time points and number of longitudinal variables	0.496277
In the case of an Elman-network as an encoder: Activation function of the hidden layers	
Dimension of initial condition (ignoring static features) of the latent ODE in percent of the number of longitudinal variables	1.20947
Number of hidden units of the feed-forward network representing the right-hand side of the latent ODE in percent of the dimension of the initial conditions (ignoring static features) of the Neural latent ODE	3.545
Steps to solve in the latent ODE	2
Activation function of the feed-forward network representing the right-hand side of the latent ODE	Identity
Number of hidden units in the decoder network, expressed as percent of the product of number of timepoints and the number of longitudinal variables	0.932988
Activation function of the decoder	TanH
Fraction of input drop-out units in the decoder	0.0961177
Number of mixture components in the Gaussian Mixture Model	6
Dimension of the latent representation of the static variables	3
Weighting parameter in MultiNODE ELBO	0.312058
Number of trainable parameters	2497

SIR noise 75%

Learning rate	0.00417377
Number of epochs	208
Batch size as fraction of the number of patients	0.99479
Encoder for longitudinal variables	Elman-network
Number of hidden units of the encoder in percentage of the product of number of time points and number of longitudinal variables	0.502702
In the case of an Elman-network as an encoder:	TanH

Activation function of the hidden layers	
Dimension of initial condition (ignoring static features) of the latent ODE in percent of the number of longitudinal variables	1.21916
Number of hidden units of the feed-forward network representing the right-hand side of the latent ODE in percent of the dimension of the initial conditions (ignoring static features) of the Neural latent ODE	3.1724
Steps to solve in the latent ODE	4
Activation function of the feed-forward network representing the right-hand side of the latent ODE	TanH
Number of hidden units in the decoder network, expressed as percent of the product of number of timepoints and the number of longitudinal variables	0.277881
Activation function of the decoder	Identity
Fraction of input drop-out units in the decoder	0.0178918
Number of mixture components in the Gaussian Mixture Model	2
Dimension of the latent representation of the static variables	3
Weighting parameter in MultiNODE ELBO	0.933758
Number of trainable parameters	208

SIR noise 100%

Learning rate	0.00123073
Number of epochs	508
Batch size as fraction of the number of patients	0.199226
Encoder for longitudinal variables	Elman-network
Number of hidden units of the encoder in percentage of the product of number of time points and number of longitudinal variables	0.79226
In the case of an Elman-network as an encoder: Activation function of the hidden layers	ReLU
Dimension of initial condition (ignoring static features) of the latent ODE in percent of the number of longitudinal variables	3.05187
Number of hidden units of the feed-forward network representing the right-hand side of the latent ODE in percent of the dimension of the initial conditions (ignoring static features) of the Neural latent ODE	2.7973
Steps to solve in the latent ODE	2
Activation function of the feed-forward network representing the right-hand side of the latent ODE	ReLU
Number of hidden units in the decoder network, expressed as percent of the product of number of timepoints and the number of longitudinal variables	0.277881

Activation function of the decoder	Identity
Fraction of input drop-out units in the decoder	0.95484
Number of mixture components in the Gaussian Mixture Model	6
Dimension of the latent representation of the static variables	5
Weighting parameter in MultiNODE ELBO	1.20909
Number of trainable parameters	4133

Supplementary References:

1. Snoek, J., Larochelle, H., & Adams, R. P. (2012). Practical bayesian optimization of machine learning algorithms. *Advances in neural information processing systems*, *25*.
2. Takuya Akiba, Shotaro Sano, Toshihiko Yanase, Takeru Ohta,and Masanori Koyama. 2019. Optuna: A Next-generation Hyperparameter Optimization Framework. In KDD.
3. Annalisa Buniello u. a. „The NHGRI-EBI GWAS Catalog of published genome-wide association studies, targeted arrays and summary statistics 2019“. In: Nucleic Acids Research 47 (D1 8. Jan. 2019), S. D1005–D1012. ISSN: 0305-1048, 1362-4962. DOI: 10.1093/nar/gky1120.
