# Supplementary material for: Generation of realistic synthetic data using Multimodal Neural Ordinary Differential Equations
Source: NPJ Digit Med. 2022 Aug 20;5:122. doi: 10.1038/s41746-022-00666-x (PMC9391444; doi:10.1038/s41746-022-00666-x)
Supplement: Supplementary file 2 — Reporting Summary [file 41746_2022_666_MOESM2_ESM.pdf]

nature portfolio

Corresponding author(s): Holger Fröhlich

Last updated by author(s): May 9, 2022

Reporting Summary

Nature Portfolio wishes to improve the reproducibility of the work that we publish. This form provides structure for consistency and transparency in reporting. For further information on Nature Portfolio policies, see our Editorial Policies and the Editorial Policy Checklist.

Statistics

For all statistical analyses, confirm that the following items are present in the figure legend, table legend, main text, or Methods section.

n/a	Confirmed	
☐	☒	The exact sample size (*n*) for each experimental group/condition, given as a discrete number and unit of measurement
☐	☒	A statement on whether measurements were taken from distinct samples or whether the same sample was measured repeatedly
☐	☒	The statistical test(s) used AND whether they are one- or two-sided<br>*Only common tests should be described solely by name; describe more complex techniques in the Methods section.*
☐	☒	A description of all covariates tested
☐	☒	A description of any assumptions or corrections, such as tests of normality and adjustment for multiple comparisons
☐	☒	A full description of the statistical parameters including central tendency (e.g. means) or other basic estimates (e.g. regression coefficient) AND variation (e.g. standard deviation) or associated estimates of uncertainty (e.g. confidence intervals)
☒	☐	For null hypothesis testing, the test statistic (e.g. *F*, *t*, *r*) with confidence intervals, effect sizes, degrees of freedom and *P* value noted<br>*Give P values as exact values whenever suitable.*
☒	☐	For Bayesian analysis, information on the choice of priors and Markov chain Monte Carlo settings
☒	☐	For hierarchical and complex designs, identification of the appropriate level for tests and full reporting of outcomes
☒	☐	Estimates of effect sizes (e.g. Cohen's *d*, Pearson's *r*), indicating how they were calculated

Our web collection on statistics for biologists contains articles on many of the points above.

Software and code

Policy information about availability of computer code

Data collection	No code was used to collect data data.
Data analysis	All used code was made available at https://github.com/philippwendland/MultiNODEs

For manuscripts utilizing custom algorithms or software that are central to the research but not yet described in published literature, software must be made available to editors and reviewers. We strongly encourage code deposition in a community repository (e.g. GitHub). See the Nature Portfolio guidelines for submitting code & software for further information.

Data

Policy information about availability of data

All manuscripts must include a data availability statement. This statement should provide the following information, where applicable:

- Accession codes, unique identifiers, or web links for publicly available datasets
- A description of any restrictions on data availability
- For clinical datasets or third party data, please ensure that the statement adheres to our policy

The PPMI dataset is available at: https://www.ppmi-info.org. The NACC dataset is available at : https://www.naccdata.org. The data is shared by the data owners after successful application. Both studies retrieved informed consent for data sharing form their participants and follow the declaration of Helsinki to ensure ethical data collection.

Field-specific reporting

Please select the one below that is the best fit for your research. If you are not sure, read the appropriate sections before making your selection.

☒ Life sciences ☐ Behavioural & social sciences ☐ Ecological, evolutionary & environmental sciences

For a reference copy of the document with all sections, see nature.com/documents/nr-reporting-summary-flat.pdf

Life sciences study design

All studies must disclose on these points even when the disclosure is negative.

Sample size	NACC: 2284<br>PPMI: 354
Data exclusions	We analyzed only the de-novo Parkinson's disease patients from PPMI to ensure that the diagnosis/disease trajectories were roughly aligned in time for all participants.<br>For NACC, we took only patients that received their diagnosis during the runtime of the study and aligned them based on that diagnosis for the same reason stated above.
Replication	We used 2 independet real world datasets and one simulated dataset to showcase our modelling framework. Across most assessments the results were in concordance. The only exception being that NACC data was more difficult to learn for the model whihc might be due to the complexity of the underlying time dependent process.
Randomization	When optimizing hyperparameters, patients were sorted into training and test data using a cross-validation split.
Blinding	Per dataset only one group was used for model training (ie. all participants were used). For data analysis, groups were not blinded but predefined algorithms and statistical procedures were used.

Reporting for specific materials, systems and methods

We require information from authors about some types of materials, experimental systems and methods used in many studies. Here, indicate whether each material, system or method listed is relevant to your study. If you are not sure if a list item applies to your research, read the appropriate section before selecting a response.

Materials & experimental systems

n/a	Involved in the study	
☒	☐	Antibodies
☒	☐	Eukaryotic cell lines
☒	☐	Palaeontology and archaeology
☒	☐	Animals and other organisms
☐	☒	Human research participants
☒	☐	Clinical data
☒	☐	Dual use research of concern

Methods

n/a	Involved in the study	
☒	☐	ChIP-seq
☒	☐	Flow cytometry
☒	☐	MRI-based neuroimaging

Human research participants

Policy information about studies involving human research participants

Population characteristics	In this study, all variables can be considered to be outcomes and thus no real covariates were present. All relevant summary statistics describing the synthetic and real data are provided and discussed elaborately in the manuscript and can be found in the form of figures at https://github.com/philippwendland/MultiNODEs.
Recruitment	The patients were not recruited by the authors of this study but the data owners. All information can be found on the websites named in the data availability statement above.
Ethics oversight	Ethical approval for data collection was granted to the data owners who collected the data.

Note that full information on the approval of the study protocol must also be provided in the manuscript.
